# Antimicrobial and cytotoxic capacity of pyroligneous extracts films of *Eucalyptus grandis* and chitosan for oral applications

**DOI:** 10.1038/s41598-021-00529-7

**Published:** 2021-11-02

**Authors:** Juliana Leitzke Santos de Souza, Tomaz Alves, Laísa Camerini, Fernanda Nedel, Angela Diniz Campos, Rafael Guerra Lund

**Affiliations:** 1 Post-Graduate Program in Biochemistry and Bioprospecting, Federal University of Pelotas, Eliseu Maciel Avenue, Building 31, Pelotas, RS 96010-900 Brazil; 2 Graduate Program in Oral and Craniofacial Biomedicine, University of North Carolina at Chapel Hill, 385 S Columbia St, Chapel Hill, NC 27599 USA; 3 Post-Graduate Program in Health and Behavior, Catholic University of Pelotas, 373 Gonçalves Chaves Street, Room 411C, Pelotas, RS 96015-560 Brazil; 4 Post-Graduate Program in Health and Behavior, Catholic University of Pelotas, 373 Gonçalves Chaves Street, Room 411C, Pelotas, RS 96015-560 Brazil; 5 Brazilian Agricultural Research Corporation, Embrapa Temperate Climate, Highway BR-392, 78th km, 9th district, Monte Bonito, Pelotas, RS 96010-971 Brazil; 6 Post-Graduate Program in Biochemistry and Bioprospecting, Laboratory of Oral Microbiology, Pelotas Dental School, Federal University of Pelotas, 457 Gonçalves Chaves, Room 503, Pelotas, RS 96015-560 Brazil

**Keywords:** Microbiology, Materials science

## Abstract

Chitosan films containing distilled pyroligneous extracts of *Eucalyptus grandis* (DPEC), characterized and developed by Brazilian Agricultural Research Corporation—Embrapa Temperate Agriculture (EMBRAPA-CPACT), were evaluated for antimicrobial activity against *Candida albicans*, *Streptococcus mutans*, and *Lactobacillus acidophilus* by direct contact test. Further, their capacity for the prevention of teeth enamel demineralization and cytotoxicity in vitro were also determined. The natural polymers were tested at different concentrations (1500–7500 µg mL^−1^) and the formulation of an experimental fluoride varnish with antimicrobial activity was evaluated by direct contact test, whereas cytotoxicity was analyzed through the colorimetric MTT assay. Preliminary data showed no statistically significant differences in cytotoxicity to NIH/3T3 cell line when DPEC is compared to the control group. On the other hand, the antimicrobial capacity and demineralization effects were found between the test groups at the different concentrations tested. Chitosan films containing distilled pyroligneous extracts of *E. grandis* may be an effective control strategy to prevent biofilm formation related to dental caries when applied as a protective varnish. They may inhibit the colonization of oral microorganisms and possibly control dental caries through a decrease in pH and impairment of enamel demineralization.

## Introduction

Several oral health conditions are acknowledged as public health issues because of their prevalence, severity, and individual and community impact, which entail costs to the health system and the existence of effective methods of prevention and treatment. Dental caries is an important pathology with widespread and worldwide incidence affecting individuals of all age groups^1,2^. Several bacterial species have been associated with the various degrees of dental caries, including *Streptococcus mutans, Lactobacillus acidophilus, Pseudomonas aeruginosa, Actinomyces viscosus and Staphylococcus aureus*^3–5^. Among these microorganisms, *S. mutans* and *L. acidophilus* seem to play a significant role in the cariogenic processes^6^ and have been pointed as one of the most prevalent biofilm species when compared to others^7^.

The symbiotic relationship between bacteria and fungal species in biofilms provides important substrates, metabolites and growth factors in certain circumstances^8^ and these interactions or ‘communication’ mechanisms between different species that live in biofilms, occur when microorganisms exchange chemical signals known as quorum sensing^9^. *Candida albicans* is a well-known example of a microbial agent that is frequently harbored as a commensal item of the healthy human mucosa. In fact, it may behave locally and systemically as an opportunistic pathogen. Candidiasis is the most common opportunistic fungal infection affecting the oral mucosa^10^ and enough evidence indicates that *Candida* species have a crucial role in caries formation processes. Consequently, oral levels of *Candida* have been frequently used as indicators of caries activity^11–14^.

Several antimicrobial agents have been widely used for oral disease therapies. However, side effects and the increasing occurrence of drug resistance-associated outcomes caused by currently available medications justify the search for new natural antimicrobial agents that are capable of targeting oral pathogens and providing safety usage to patients^15^.

Distilled pyroligneous extract (DPE) is a complex mixture of compounds derived from the condensation of smoke generated by wood carbonization^16^. DPE has applications in several areas and possesses antimicrobial, antioxidant and anti-inflammatory functions. It is also capable of imparting organoleptic and antimicrobial properties to smoked food^16–19^.

Several species of *Eucalyptus*, belonging to the Myrtaceae family of dicotyledonous plants, are among the most commonly cultivated plantation trees worldwide, and their wood may be used for multiple purposes, including veneer, firewood and the production of essential oils^20^. Further, the genus *Eucalyptus* has a long usage history in traditional medicine and has been attracting great interest worldwide for its antibacterial, antiviral, antifungal, anti-inflammatory and insect-repellent properties for cosmetic, pharmaceutical, nutraceutical and furniture purposes^21^.

Chitosan is a natural mucoadhesive polymer extract from chitin, characterized as a cationic polymer capable of intense electrostatic interaction with negatively charged mucosa through the salt bridge effect^22,23^. It has antibacterial and antifungal properties and is used for food protection^24,25^. Due to its biocompatible and functional properties, chitosan also demonstrates a broad potential for its application as a biomaterial. Mucoadhesive buccal films based on chitosan may be an alternative dosage form for oral diseases in children^26^. Additionally, chitosan may serve as a barrier against acid penetration, contributing to its demineralization inhibition in the process of tooth enamel demineralization and inhibiting the release of mineral components to the oral cavity^27^. Moreover, antimicrobial films may be prepared with chitosan blended with natural extracts^24,28^. One of the main advantages displayed by oral drug delivery systems is the capability of direct local release of potential pharmaceuticals capable of treating tooth-related diseases, fungal infections, gingivitis and periodontitis^29^.

Patent formulations were registered as green chemistry materials at the National Institute of Intellectual Property (Instituto Nacional de Propriedade Intelectual) in Brazil (PCT/BR2013/000597), USA (US20150336854 A1) and Germany (DE112013006230T5) as a phytoprotector for use in agriculture^30^. Results of current research have been registered at the INPI (BR102012033149-7) for use as oral antimicrobial materials. In this context, the originality of this study is the introduction of pyroligneous extracts in the composition of chitosan films for topical use in the oral cavity.

Therefore, this study aimed to evaluate the antimicrobial activity of chitosan films containing DPEC against *Candida albicans*, *Streptococcus mutans*, and *Lactobacillus acidophilus* by direct contact test, as well as their capacity for the prevention of teeth enamel demineralization and in vitro cytotoxicity.

## Materials and methods

### Characterization of extracts

The pyroligneous extraction and distillation procedures were performed as previously described^30^. Chitosan was supplied by Nutrifarm, with a 97% degree of deacetylation, determined by proton magnetic resonance^30^. The eucalyptus plantation is located at Embrapa Temperate Climate Experimental Station, in Pelotas RS Brazil (31° 40′ 49″ S; 52° 26′ 18″ W, at 60 m above sea level), season 2013/2014. According to Köppen classification, the region´s climate is Cfa, temperate and humid, with hot summers.

The chitosan films containing pyroligneous extract were developed by the Brazilian Agricultural Research Corporation—Embrapa Temperate Agriculture (EMBRAPA-CPACT). Briefly, smoke and gases were collected at an 80–150 °C temperature range. The pyrolysis extract was stored for 6 months for stabilization. The product was then distilled in a Buchi rotary evaporator (model R-114) and the fraction was collected at a temperature ranging between 60 and 75 °C and labeled DPE (namely DPL = distilled pyroligneous liquor)^30^. Table 1 shows the main chemical compounds identified in the DPE, whilst Table 2 presents the characterizations of films in each tested group. Preparations and structural characterizations [X-ray diffraction (XRD); scanning electron microscopy (SEM); Fourier transform infrared spectroscopy (FTIR)] of these formulations of distilled pyroligneous extract, obtained from *Eucalyptus grandis*, and chitosan, were previously published^19,27^.

**Table 1:** Main chemical compounds identified in the distilled pyroligneous extract.

Identified Compound	
1	Tetrahydropyran
2	3-Methyl-2-cyclopenten-1-one
3	Phenol
4	2,3-Dimethyl-2-cyclopenen-1-one
5	Phenol,2-methyl (ortho-cresol)
6	Phenol,3-methyl (meta-cresol)
7	Phenol,2-methoxy
8	Phenol,2,3-dimethyl (2,3-xylenol)
9	Para-cresol,2-methoxy
10	Phenol,4-ethyl-2-methoxy
11	Phenol-2,6-dimethoxy

*Based on mass spectroscopy analysis (Porto et al.^30^).

**Table 2:** Description of films by chitosan containing pyroligneous extract in each group tested.

Characterizations of groups	Abbreviation
Distilled pyroligneous extract + 3% chitosan	DPEC 3%
Distilled pyroligneous extract + 1% chitosan	DPEC 1%
Distilled pyroligneous extract + 3% chitosan + Cu 2 mg/mL	DPEC 3% + Cu
Distilled pyroligneous extract + 3% chitosan + Si 0.002 mg/mL	DPEC 3% + Si
Distilled pyroligneous extract + 3% chitosan + NaF 5%	DPEC 3% + NaF 5%
Distilled pyroligneous extract (Control)	DPE
Duraphat, Colgate-Palmolive, USA (Commercial varnish, Positive Control)	NaF 5%
Negative control (no treatment)	Control

### Antimicrobial assays

#### Direct contact test

The direct contact test was conducted as previously described^16^. Three bacterial species were tested, namely, *C. albicans* (ATCC 62342), *S. mutans* (ATCC 25175) and *L. acidophilus* (ATCC4356), obtained from the Oswaldo Cruz Foundation collection. At 24 h before the microbial challenge, species were subcultured overnight in Sabouraud dextrose agar (SD; Difco) for *C. albicans*; brain heart infusion (BHI) agar (Difco) for *S. mutans*; and Lactobacilli MRS (MRS) agar (Difco) for *L. acidophilus*, and then incubated at 37 °C. The inoculum was prepared according to the 0.5 MacFarland scale (1 × 10^8^ CFU/mL): a 10 μL-aliquot was suspended in 96 well plates containing five concentrations of the film groups (Table 2) ranging from 1500 to 7500 μg/mL. The plates were incubated for 1 and 24 h. Subsequently, 240 μL of SD broth, BHI, and MRS broth (Difco) were added to each well, and the plates were agitated by a plate shaker for 5 min, at 150 rpm. Serial tenfold dilutions of 100 µL sample in 900 µL of broth were then made. Further, four 25 μL aliquots were plated onto 9 cm-Petri dishes containing Sabouraud, BHI or MRS agar and incubated at 37 °C for 48 h. After this period, the number of colony-forming units was determined by two trained examiners. Positive (inoculum without the presence of any product) and negative (only the culture medium without microorganisms) controls were established for each group. All the experiments were performed twice and samples were organized in triplicates. In order to calculate the bacterial growth inhibition, we used the following formula where BGI % is bacterial growth inhibition percentage, Ac is an average of six replicates of colony-forming unit (CFU) counts for the negative controls, and At is an average of 6 replicates of CFU counts for samples from the other groups:

$${\text{BGI}}\% = \left[ {\left( {{\text{Ac}}{-}{\text{At}}} \right)/{\text{Ac}}} \right] \times {1}00$$

#### Biofilm microcosms: experimental design and conditions

The microcosm biofilm model was carried out according to previous reports^31^. In fact, the biofilm model is one of the closest simulations to in vivo models, making it possible to more accurately reproduce the complexity of a real dental biofilm in vitro^32–34^. The use of saliva or dental plaque as an inoculum for biofilm formation in microtiter plates is certainly the most commonly used method^35^. Human saliva was used as the inoculum with bovine enamel as substratum. The nutrient growth medium used for the experiments was a defined medium enriched with mucin (DMM; pH 6.8). Ethical approval was granted by the Ethics Committee, School of Dentistry, Federal University of Pelotas, Protocol No. 66039317.3.0000.5318 (Pelotas, RS, Brazil). The study was performed according to the guidelines of the Declaration of Helsinki. All the experiments were performed twice and samples were organized in triplicates. The outcome variables assessed were pH from the medium supernatant and mineral loss evaluated by the percentage surface hardness change (%SHC)^31^.

#### Enamel disks

A cylindrical diamond-coated drill (trephine), perpendicular to the buccal surface of freshly extracted bovine central incisors, was employed, and enamel disks (5 mm diameter and 2 mm thickness) were obtained^36^. Dentine and enamel surfaces were wet-ground with 600 and 600/800/1200/1500/2000-grit silicon carbide papers, respectively. Both surfaces were plane-parallel. Nail varnish was applied on the sides and bottom of the disks, leaving only the buccal enamel surface exposed. The disks were fixed with a holder prepared with orthodontic wire and kept in a vertical position during the experimental procedures. They were sterilized by gamma radiation at 4.08 KGy (Eldorado 78; Best Theratronics) and kept at 4 °C in a humid atmosphere until use. All the experiments were performed twice and samples were organized in triplicates.

#### Saliva collection

For each experimental run, fresh stimulated saliva was collected from a healthy subject (a 20-year-old female) who had not been under antibiotic therapy for at least 6 months. Saliva was collected in the morning (during fasting), and the volunteer abstained from oral hygiene for 24 h prior to collection^36^. The volunteer signed an informed consent for study participation.

#### Treatment of chitosan films containing pyroligneous extract

A 10% sucrose solution was used as a cariogenic challenge for all groups (6 h per day)^37,38^. About 7500 µg of each experimental film, described in Table 2, was used for antimicrobial and anti-cariogenic treatment. The film was applied on the first day using a microbrush and then dried with a jet of air. Negative control received no treatment, and a commercial varnish containing fluoride (5% NaF varnish-Duraphat, Colgate-Palmolive) was used as a positive control.

#### Inoculation procedure and biofilm growth

Enamel disks were transferred aseptically into sterile wells (24-well tissue culture plate; Techno Plastic Products, Trasadingen, Switzerland) and 0.4 mL of fresh homogenized saliva was dispensed onto each enamel disk. After 1 h at room temperature (22 ± 3 °C), saliva was aspirated, and 1.8 mL of growth medium was added^36^. After the addition of sucrose, disks were dip-washed for 10 s in sterile saline solution and transferred to a new plate with DMM. Plates were incubated in 5–10% CO_2_ and 51% O_2_ (Anaerobac–Probac) in anaerobic jars (Anaerobac–Probac) for up to 5 days at 37 °C, without shaking.

Growth medium was replaced daily, twice a day (6 h/DMM + 10% sucrose and 18 h/DMM), in all experiments. Prior to medium replacement, plates were gently shaken; the disks were dip-washed for 10 s in sterile saline and transferred to a new plate where fresh medium was added. At the end of the experimental periods, the disks were sonicated in 1 mL of 0.9% NaCl for microbiological assays, carefully cleaned with a soft bristle toothbrush and distilled water, and kept at 4 °C in a humid atmosphere in microtubes until enamel hardness analysis was performed.

#### Bacterial viability

A 100 µL aliquot of the sonicated suspension was diluted in 0.9% NaCl, and serial dilutions were inoculated in duplicate (20 µL drops) in the following culture media: blood agar for total anaerobic microbiota (MT) culture; Mitis Salivarius Agar for the determination of total streptococci (ST); agar Mitis Salivarius (Bacitracin MSB) for counting mutans streptococci (SM), and Rogosa agar for *Lactobacillus* counts. Plates were then incubated in anaerobiosis (96 h)^39^. CFUs were quantified, and the results were expressed as CFU/mg dry weight of the biofilm^37^.

#### Biofilm supernatant pH readings

After growth medium replacements, pH was individually recorded from each well (Quimis 50w-Quimis; V621 electrode–Analion,) twice a day. Readings were obtained after the period under DMM (18 h) and after the period under DMM with sucrose (6 h)^36^.

### Prevention of teeth enamel demineralization

#### Enamel hardness

The hardness of the enamel disks was tested by six indentations, spaced 100 µm from each other, with a Knoop diamond, loaded with a 25 g weight, for 5 s (Micro Hardness Tester FM 700–Future-Tech Corp.)^36^. Surface hardness reading was performed before (sound enamel; SH) and after experiments (SH2).

The percentage of SHC was calculated by the following equation:

$$\left[ {\% {\text{SHC}} = {1}00\left( {{\text{SH2}} - {\text{SH}}} \right)/{\text{SH}}} \right]$$

### Cytotoxicity assay

#### Cell culture

Mouse fibroblasts of NIH/3T3 cells were obtained from the Rio de Janeiro Cell Bank (PABCAM, Federal University of Rio de Janeiro). The cells were cultured in Dulbecco’s modified eagle medium (DMEM) supplemented with 10% fetal bovine serum (FBS); DMEM and FBS were purchased from Vitrocell Embriolife and Gibco, respectively. Cells were grown at 37 °C in an atmosphere with 95% humidified air and 5% CO. The experiments were performed with cells in the logarithmic growth phase.

#### Determination of cytotoxicity

The viability of NIH/3T3 cells was determined by measuring the reduction of soluble MTT [3-(4,5-dimethylthiazol-2-yl)-2,5-diphenyltetrazolium bromide] to water-insoluble formazan by the colorimetric assay^40^. Cells were then seeded at a density of 2 × 10^4^ cells per well in a volume of 100 µL in 96-well plates and grown at 37 °C in a humidified atmosphere of 5% CO_2_ and 95% air for 24 h before use in MTT assay. Cells were incubated with two different concentrations of the film groups (1500 and 7500 μg/mL) of films, for 48 h. These compounds were dissolved in DMSO and added to DMEM, supplemented with 10% FBS at the desired concentrations. Final DMSO concentration in the culture medium never exceeded 0.5%, and a control group exposed to an equivalent concentration of DMSO was evaluated. After incubation, 20 µL of MTT (5 mg MTT/mL solution) were added to each well. The plates were incubated for an additional 3 h, and the medium was discarded. 200 µL of DMSO were added to each well, and formazan was solubilized on a shaker for 5 min. The absorbance of each well was read on a microplate reader (Thermo Plate TP-Reader, Thermo Fisher Scientific) at 492 nm. The percentage inhibition of cell growth was determined as follows: inhibitory rate = (1 − Abs492treated cells/Abs492control cells) × 100. All observations were validated by at least two independent experiments and analyses for each experiment were performed in triplicate.

### Statistical analysis

Results of cytotoxicity were analyzed by Kruskal–Wallis one-way ANOVA on ranks. Differences among groups were significant at p < 0.001. Statistical analyses were performed using SigmaPlot version 12.0 (Systat Software, Inc., San Jose California USA).

## Results

### Antimicrobial assays

Formulations of chitosan films containing pyroligneous extract were evaluated for their in vitro activity against *C. albicans, S. mutans* and *L. acidophilus*, using the direct contact method. Results demonstrated the films’ ability to inhibit the growth of the microorganisms at low concentrations and possibly prevent and treat oral infections caused by them. The current study assessed five formulations containing pyroligneous extract and chitosan, one pure pyroligneous extract control and one commercial fluorine varnish (5% NaF varnish).

Table 3 shows the films’ inhibition potential at 1 and 24 h after exposure to *C. albicans* (A), *S. mutans* (B) and *L. acidophilus* (C), in vitro, using the direct contact assay. After a 24 h-exposure, all films with pyroligneous extract reduced microbial adherence for all species tested, demonstrating that the films are effective anti-biofilm agents.

**Table 3:** Inhibition percentage (%) of DPEC on *C. albicans*(A), *S. mutans*(B) and *L. acidophilus* (C) (respectively) exposed for 1 and 24 h of direct contact with the concentration ranging from 1500 μg mL^−1^ to 7500 μg mL^−1^ of film groups.

	Formulation																		
Time	1 h									24 h									
Concentration(µg mL^−1^)	7500		6000		4500	3000		1500		7500		6000		4500		3000		1500	
**A**																			
DPEC 3%	24		32		23	13		45		**100**		**100**		**100**		**100**		**100**	
DPEC 1%	50		21		24	43		46		**100**		**100**		**100**		**100**		**100**	
DPEC 3% + Cu	100		99		100	74		100		**100**		**100**		**100**		**100**		**100**	
DPEC 3% + Si	19		4		9	44		45		**100**		**100**		**100**		**100**		**100**	
DPEC 3% + NaF 5%	27		96		78	67		32		**100**		**100**		**100**		**100**		**100**	
DPE	86		86		91	56		32		**100**		**100**		**100**		**100**		**100**	
NaF 5%	0		–		–	–		–		**100**		–		–		–		–	
**B**																			
DPEC 3%	98.8	95.1		90.5			99.5		98.7		**100**		**100**		**100**		**100**		**100**
DPEC 1%	97.2	99.3		99.2			99.4		94.7		**100**		**100**		**100**		**100**		**100**
DPEC 3% + Cu	97.7	99.9		99.8			99.9		99.6		**100**		**99.9**		**100**		**100**		**100**
DPEC 3% + Si	94.2	91.8		93.7			93.8		74.6		**100**		**99.8**		**100**		**100**		**100**
DPEC 3% + NaF 5%	100	100		100			100		100		**100**		**100**		**100**		**100**		**100**
DPE	94.6	93.0		91.3			91.9		59.5		**100**		**100**		**99.9**		**99.9**		**100**
NaF 5%	100	–		–			–		–		**100**		–		–		–		–
**C**																			
DPEC 3%	29.2	93.4		NI			10.8		19.9		**100**		**100**		**100**		**100**		**100**
DPEC 1%	81.8	71.1		42.3			61.7		56.9		**100**		**100**		**100**		**100**		**100**
DPEC 3% + Cu	92.0	98.9		98.5			90.1		66.3		**100**		**100**		**100**		**100**		**100**
DPEC 3% + Si	32.7	NI		NI			17.0		NI		**100**		**100**		**100**		**100**		**100**
DPEC 3% + NaF 5%	87.0	93		86			87		90		**100**		**100**		**100**		**100**		**100**
DPE	52.0	30.3		NI			NI		NI		**100**		**100**		**100**		**100**		**100**
NaF 5%	100	–		–			–		–		**100**		–		–		–		–

Table 4 shows the antibacterial effect of DPEC films in the biofilm microcosms test. There was no statistically significant difference between films when compared to control (p < 0.05). The same occurred in aciduric microorganisms, *Lactobacilli* and *S. mutans*. In the case of *Lactobacilli*, only DPEC 3% was statistically significantly different from control (p < 0.05).

**Table 4:** Bacterial viability (log CFU mg^−1^) for biofilm microcosms.

Group	Lactobacillus	Total streptococci	Mutans streptococci	Total anaerobic
DPE	6. (± 0.4)^a^	6.5 (± 0.4)^a^	4.5 (± 1.2)^a^	8.9 (± 0.2)^a^
DPEC 1%	5.7 (± 1.2)^a^	5.6 (± 1.2)^a^	4.2 (± 0.6)^a^	8.8 (± 0.2)^a^
DPEC 3%	5.4 (± 1.2)^b^	5.5 (± 1.1)^a^	4.3 (± 0.6)^a^	8.9 (± 0.2)^a^
DPEC 3% + Fluoride	6.1 (± 0.8)^a^	6.3 (± 0.6)^a^	4.9 (± 1.6)^a^	8.6 (± 0.6)^a^
Commercial Varnish	5.7 (± 1.1)^a^	6.1 (± 0.6)^a^	4.5 (± 1.7)^a^	8.6 (± 0.6)^a^
Control	6.2 (± 0.2)^a^	6.3 (± 0.5)^a^	3.5 (± 0.5)^a^	8.6 (± 0.4)^a^

Similar letter indicates no statistically significant differences in CFU counts between the groups tested. A value of *p* ≤ 0.05 was considered significant (Tukey’s test).

Table 5 demonstrates the biofilm supernatant pH readings obtained after the period under DMM (18 h) and after the period under DMM with sucrose (6 h) for 5 days. Changes have been given according to sucrose addition in the cariogenic challenge.

**Table 5:** Biofilm supernatant pH readings.

Time	6 h	18 h
**Group**		
DPE	5.0	7.0
DPEC 1%	5.0	7.0
DPEC 3%	5.0	7.0
DPEC 3% + NaF	5.0	7.0
Commercial varnish	5.0	7.0
Control	5.0	7.0

### Prevention of teeth enamel demineralization

**Figure 1:**
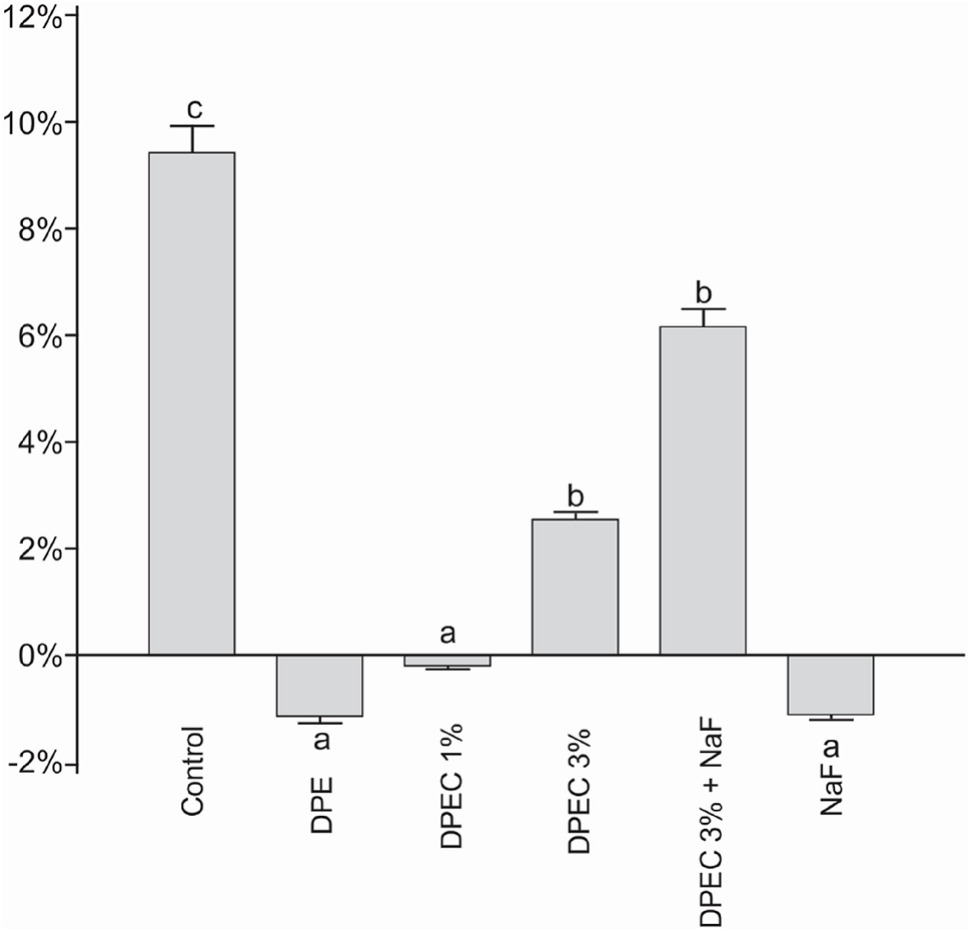
Mean rates (n = 10) and SD of the percentage surface hardness change (%SHC) for chitosan film containing pyroligneous extract treatments and control (saline solution). Enamel protection is indicated by negative rates of %SHC. Distinct letters show statistically different %SHC (p ≤ 0.05) among treatments.

The percentage of enamel hardness (% SHC; Fig. 1) of biofilm polymer films exposed for 5 days at a concentration of 7500 μg/mL of DPEC was analyzed. DPE and DPEC 1% exhibited their ability to protect tooth enamel against demineralization when compared to other groups, including commercial varnish.

### Cytotoxicity assay

**Figure 2:**
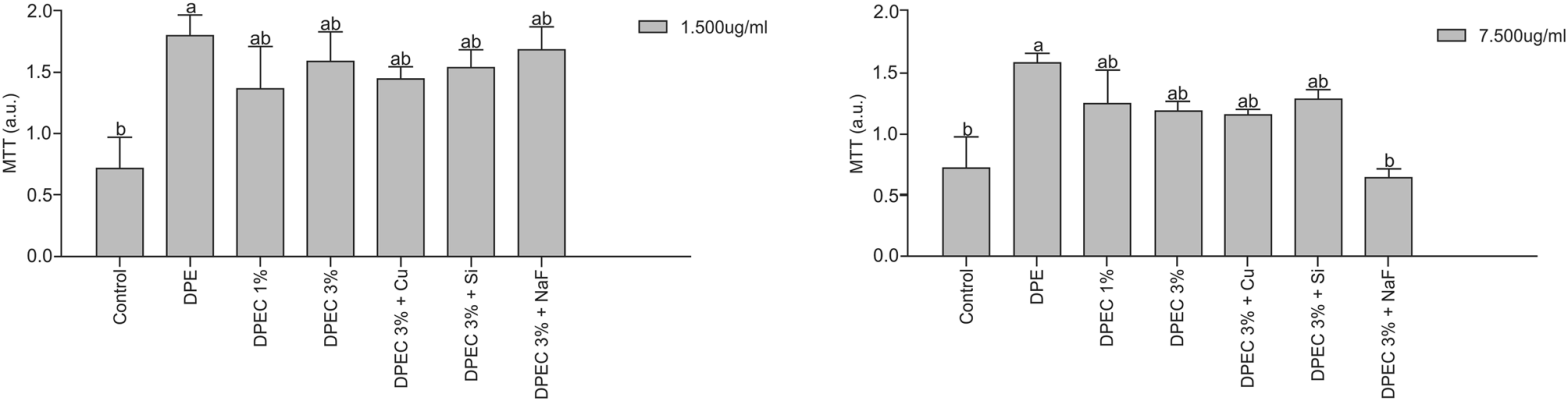
Effect on cell viability of NIH/3T3 cell line exposed to 24 h of contact at 1500 and 7500 μg/mL DPEC films. Data are expressed as means ± SD in arbitrary units (a.u.-absorbance at 492 nm). Different letters indicate statistically significant differences between compounds tested. Rate of p ≤ 0.05 was considered significant by Tukey’s test).

The colorimetric assay MTT (3-[4,5-dimethyl-thiazol-2-yl]-2,5-diphenyltetrazolium bromide) was performed to evaluate whether the tested compounds may alter cell viability. At 1500 and 7500 μg/mL (Fig. 2), the test compounds did not show cytotoxicity against NIH/3T3 cell line, whilst DPE compound revealed increased cell viability (p = 0.03–1500 μg/mL, p = 0.001–7500 μg/mL). Thus, the DPEC films did not present cytotoxicity against this cell line at concentrations in which it demonstrated antimicrobial activity.

### Ethics declarations

Methods were carried out following the Helsinki declaration and the regulatory guidelines and norms obey the 466/12 resolution for research ethics in Brazil. The Research Ethics Committee of the Pelotas Dental School Federal University of Pelotas approved this research (Protocol No. 66039317.3.0000.5318). Volunteer who agreed to donate saliva in this study signed informed consent, authorizing their participation.

## Discussion

All formulations of DPEC films showed a bactericidal effect in the antimicrobial evaluation through the direct Contact test and a 100% growth inhibition after a 24 h period. Therefore, the formulation seems to be advantageous for use as an oral antimicrobial treatment. DPE alone has not been taken into account because it does not form a film and it is used only as a control in the tests.

Results corroborate previous findings on the antimicrobial activity of representative natural low flavor antimicrobial pyroligneous extract derivatives described by the US patent 20070212310 A1. In fact, they exhibit potent antibiotic activity against pathogenic bacteria of the oral cavity. Other pyroligneous extracts were previously tested concerning their ability to inhibit the growth of various bacterial species known to be relevant to tooth decay and/or periodontal disease. These bacterial species included *S. mutans* (dental caries), *Porphyromonas gingivalis* (periodontal disease), and *Fusobacterium nucleatum* ssp. *Polymorphum* (periodontal disease)^41^.

Mass spectroscopy analysis was performed according to Porto^30^ for each retention time for DPE used in the current study. Results revealed the presence of different chemical species, including aromatic, phenolic, carbonylic and organic compounds containing heteroatoms (Table 1). These findings were consistent with those reported in the literature^18,30^. The main chemical species present in DPE used in current assay were identified as aromatic or heteroaromatic compounds, carbonylic, phenolic, and carboxylic acids^30^.

Phenol compounds have therapeutical applications as fungicide, antiseptic and disinfectant agents^42^, with activities against a wide range of microorganisms, including several viruses. Phenol is also one of the components of commercial pyroligneous extracts (also known as “liquid smoke products” “pyroligneous acids” or “wood vinegar”)^43–45^. Cresols (*o*, *m* and *p*-cresol or 2,3 and 4-methy-phenol) are used as local antiseptics, parasiticides, disinfectants and as intestinal antiseptics^42^, and they also feature as components in liquid smoke products^43,46^. Other compounds in pyroligneous extracts, such as 4-ethyl-2-methoxy-phenol, maltol, 4-oxo-methyl-esther pentanoic acid (methyl levulinate), 2,6-dimethoxy-phenol (syringol) and its derivatives and xylenols, are used as flavoring agents^43,45,47^. However, as observed previously, antibacterial and antifungal activities of pyroligneous extracts from different origins cannot be attributed simply to a unique chemical component, but rather to a synergistic combination of several ones, particularly phenolic compounds^44^.

A previous patent (US 20070212310 A1) with pyroligneous extract consisted of a piece of chewing gum, an edible film, confectionary, dentifrice, lozenge, mouthwash, and mouth spray, at a concentration of 0 to about 6% weight per unit volume (w/v) of liquid smoke derivative^41^. However, in the current study, DPEC films were tested as a topical dental varnish for caries control using a concentration of 1500–7500 µg mL^−1^ or 0.15–0.75%. These findings corroborate with the literature, confirming the in vitro antibacterial and antifungal properties of *Eucalyptus* pyroligneous extracts^48^. Moreover, pyroligneous extract and derivatives against four pathogenic strains of *C. albicans* showed the anticandidal potential for use of *Rhizophora apiculata* pyroligneous acid as an untapped source of compounds with anti-*C. albicans* activity^49^.

Fluoride varnish compositions for temporary application and adhesion to teeth include a stable liquid or gel carrier, which remains stable and translucent during storage, and a dispersed fluoride ion source (e.g., a fluoride salt such as sodium fluoride) within the carrier to biologically supply fluoride ions to the enamel hydroxyapatite. The resinous carrier is composed of a solvent, a resin component (acidic hydrogenated wood rosin) and an acidifying component (citric acid, phosphoric acid, boric acid, malic acid and the like), the latter to reduce the pH of the liquid carrier or gel to below 5^50^. The usage of these formulations as a potential commercial varnish is due to their ability to combine with a natural polymer and the antimicrobial action of the distilled pyroligneous extract. Further, formulations may be prepared with and without fluoride, depending on the purpose: tooth protection/remineralization or dental biofilm elimination.

Dental biofilm is a complex microbial community developed on the tooth surface^51^. So that biofilm inhibition may be verified, a microcosm biofilm model was employed. The above is a current trend since it offers the advantage of coming closer to the physical–chemical, microbiological and nutrient conditions, in addition to maintaining the complexity and heterogeneity of in vivo biofilms^51^. Although DPEC films did not demonstrate effectiveness in impairing the biofilm formation process after a 5-day exposure, some DPEC films proved to be efficient in protecting the enamel from demineralization and, consequently, demonstrated the potential use in tooth enamel protection. On the other hand, the species *Candida* was not quantified in the microbial analysis of the microcosm biofilm because the saliva sample did not reveal *Candida* growth.

Preliminary results showed that the DPEC films were not cytotoxic to the NIH/3T3 cell line, and no statistically significant differences (p < 0.05) were found between the test groups, in the different concentrations tested, and between the control group. The rationale for using these film concentrations in the cytotoxicity assay comes from the knowledge that they represent the lowest and highest concentration used in the antimicrobial assay by the direct contact test. The 48-h cell exposure period was based on previous studies^52,53^.

In the case of mutagenic or cancerogenic compounds in pyroligneous extracts, specifically polycyclic aromatic hydrocarbons (PAHs), several authors have pointed out that by removing wood tar from pyroligneous extracts, the PAHs are concomitantly removed^54^. PAHs have a strong environmental impact because of their mutagenic and carcinogenic properties^55^, which makes the detection of these compounds important in processed foods, drinking water, air, etc. for safety reasons. Although liquid products from pyrolysis have been assessed to establish their acute toxicity and genotoxicity, results showed that crude pyrolysis liquids of eucalyptus wood had no mutagenic properties, because PAHs are strongly adsorbed into the pitch fraction and are not bioavailable^56^.

As a rule and taking into account current in vitro results, it appears that experimental films derived from *E. grandis* pyroligneous extract, when applied as a protective varnish, may be an effective and alternative control strategy in preventing teeth demineralization. Moreover, these experimental films might serve as adjuvants in the treatment against oral microbes, avoiding the formation of biofilm, related to dental caries. Their application could be useful, for instance, both in the prevention of occlusal caries in deciduous or permanent molars in children and the treatment of root caries lesions frequently diagnosed in the elderly. The employment of formulations containing these experimental films and fluoride would be perhaps more appropriate in patients with a high risk for caries development. However, most oral infections are caused by a broad, varied and complex microbial ecosystem and, therefore, more in-depth studies are still necessary to foreground these findings.

In summary, all DPEC film formulations showed promising results in biological in vitro assays, with no cytotoxicity against NIH/3T3 cell line and good antimicrobial potential against the main bacterial strains related to cariogenesis. Therefore, the formulations might be forwarded as an adjunct drug in the treatment of dental caries, improving the prevention of tooth demineralization.
